# Smartphone-Enabled Quantification of Potassium in Blood Plasma

**DOI:** 10.3390/s21144751

**Published:** 2021-07-12

**Authors:** Achmad Syarif Hidayat, Hideyuki Horino, Izabela I. Rzeznicka

**Affiliations:** 1 Shibaura Institute of Technology, Graduate School of Engineering and Science, 3-7-5 Koto-ku, Tokyo 135-8548, Japan; mg18501@shibaura-it.ac.jp; 2 Tohoku University, 2-1-1 Katahira, Aoba-ku, Sendai 982-8577, Japan; hideyuki.horino.a6@tohoku.ac.jp

**Keywords:** potassium quantification, blood plasma, turbidity, sodium tetraphenylborate, point-of-care testing (POCT), smartphone

## Abstract

This work describes a new method for determining K^+^ concentration, [K^+^], in blood plasma using a smartphone with a custom-built optical attachment. The method is based on turbidity measurement of blood plasma solutions in the presence of sodium tetraphenylborate, a known potassium precipitating reagent. The images obtained by a smartphone camera are analyzed by a custom image-processing algorithm which enables the transformation of the image data from RGB to HSV color space and calculation of a mean value of the light-intensity component (V). Analysis of images of blood plasma containing different amounts of K^+^ reveal a correlation between V and [K^+^]. The accuracy of the method was confirmed by comparing the results with the results obtained using commercial ion-selective electrode device (ISE) and atomic absorption spectroscopy (AAS). The accuracy of the method was within ± 0.18 mM and precision ± 0.27 mM in the [K^+^] range of 1.5–7.5 mM when using treated blood plasma calibration. Spike tests on a fresh blood plasma show good correlation of the data obtained by the smartphone method with ISE and AAS. The advantage of the method is low cost and integration with a smartphone which offers possibility to measure [K^+^] on demand and in remote areas where access to hospitals is limited.

## 1. Introduction

The potassium cation (K^+^) is important in living organisms, necessary for the correct functioning of cells, particularly in muscle and nerve tissues [1]. The concentration of K^+^, [K^+^], in the human body is in the range 35–55 mmol per kilogram of body weight and is mostly present in intracellular compartments [2]. The extracellular [K^+^] accounts for only 2% of the total blood [K^+^]. In clinical diagnostic tests, the extracellular [K^+^] in blood plasma is usually measured because of the many challenges that face attempts to measure on whole blood. The normal [K^+^] in blood plasma is maintained within 3.5–5.0 mM [3]. Potassium-rich food in the diet may severely increase plasma [K^+^] but the excess is usually excreted in the urine, and also from the skin and gastrointestinal tract [4]. However, in some pathological conditions, normal plasma [K^+^] cannot be maintained. Excess (*hyperkalemia*, [K^+^] > 5.5 mM), or deficit of K^+^ (*hypokalemia*, [K^+^] < 3.5 mM) can cause life-threatening conditions such as cardiac arrhythmias as well as cardiac arrest (at [K^+^] > 7 mM) [4,5,6,7]. In the long term, inability to maintain [K^+^] may lead to other complications, such as stroke, coronary heart disease, osteoporosis, hypertension, and increased mortality, especially in the elderly population and patients with kidney diseases [8,9]. People with no underlying medical conditions may also develop K^+^ imbalance due to poorly controlled dietary intake, microbial infection, pharmaceutical side-effects, intensive physical training, or excessive sweating [10]. Recent studies indicate that hypokalemia in US population is on the rise due to increasing deficit of potassium in agriculture products [11]. It is therefore important to monitor plasma [K^+^], ideally on-demand, which would be similar to being able to test blood glucose easily as can now be done anytime anywhere using a portable amperometric glucose sensor.

There are several methods used for quantification of [K^+^] in blood plasma [12]. The first method to be developed was colorimetric, using silver cobaltinitrite reagent which yields an orange–coloured solution in the presence of K^+^ [13]. Later, flame photometry was used for quantitative analysis of plasma [K^+^] followed by more accurate atomic absorption spectroscopy (AAS) [14,15]. The advantages of AAS is that only 0.1 mL or less of blood plasma is required [15]. However, all these methods require several steps of sample preparation, bulky analytical devices, and well-trained laboratory personnel to perform measurements. Rapid progress in electronics and thin-film technology since the 1970’s has enabled the development of portable devices for quantification of [K^+^]. These devices use an ion-selective electrode (ISE) which transduces [K^+^] to a potential difference [16]. ISE and flame photometry remain the most frequently used method used in clinical laboratories, though they suffer some accuracy problems [17]. Ion-selective optodes (ISO), with potassium ion sensitive ionophore, offer another way of [K^+^] quantification, but they have issues with pH sensitivity of chromoionophores [18]. Methods based on light scattering offer a simple way of measurement [K^+^] in blood serum with high sensitivity [19,20]. Using conventional UV-VIS spectrophotometer, Tubino et al. showed 99% agreement for quantification of blood serum [K^+^] by turbidimetric method [21].

During the last two decades, small-sized point-of-care testing (PoCT) devices have been developed for medical diagnosis, such as portable blood glucose device. PoCT devices utilizing smartphones are emerging as portable alternatives for analysis, diagnosis, and other clinical applications, since they are accessible anytime, anywhere and can be used even by non-specialist member of the public [22]. One of the most enabling technologies integrated into smartphones is the digital camera, with its complementary metal-oxide-semiconductor (CMOS) sensor. The latest CMOS models can even out-perform the charge coupled device (CCD) sensors used in conventional analytical devices [23,24]. Advances in CMOS technology, along with increases in processing speed and storage capacity, have enabled the realization of low-cost, smartphone-based analytical and imaging devices [22]. In the fields of clinical analysis and medical treatment, such devices have the potential for the monitoring of physiological parameters on-demand in real-time, outside of hospital premises, using the Internet as a platform [25]. Smartphone-enabled devices can enrich the growing field of the internet-of-things (IoT) and contribute to better health management, particularly for people in remote areas who lack easy access to a hospital. There are already several smartphone-enabled PoCT devices that have been applied to medical diagnostics. Examples include smartphone-enabled PoCT for dry eye diagnosis [26], kidney diagnosis [27], cortisol quantification [28], bilirubin diagnosis [29], detection of antibodies and RNA in blood [30,31], and detection of HIV or hepatitis [32]. Smartphone fluorescence platforms for potassium detection in urine have recently been described [33]. However, there are no thorough clinical studies that could correlate [K^+^] in urine with the actual concentration in blood in a given moment of time. Luo et al. presented a smartphone colorimetric assay for detection of K^+^ in blood serum. The device showed a good correlation with the commercial microplate reader but no correlation to traditional methods of potassium detection was made [34].

In the present study, an optical attachment to a smartphone was built and a new method for K^+^ quantification in blood plasma was developed. The results of K^+^ quantification using this method were compared with those obtained using traditional ISE device and AAS instrument.

## 2. Materials and Methods

### 2.1. Chemicals and Stock Solutions

Potassium chloride (99.99%, 204099), sodium tetraphenylborate, Na-TPB, NaB(C_6_H_5_)_4_, (ACS reagent ≥ 99.5%, T25402), potassium standard for AAS calibration (TraceCERT 1000 mg/L: K in nitric acid, 96665), and lyophilized bovine plasma in sodium citrate (P4639) were purchased from Sigma-Aldrich (St. Louis, Missouri, USA). Whole bovine blood in sodium citrate was purchased from Rockland Immunochemicals, Inc. (Limerick, PA, USA). All chemicals were used without further purification. Ultrapure water with a specific resistance of 18.2 MΩ and total organic carbon below 3 ppm was used throughout the experiments. Syringe filters (PTFE, pore size 0.2 µm) were purchased from GE Healthcare Life Science.

A 146.1 mM stock solution of Na-TPB was prepared by dissolving 2.5 g of Na-TPB in 50 mL ultrapure water and passed through a syringe filter (0.22 μm pore size) to obtain a clear stock solution. Na-TPB is a known potassium precipitating agent [35]. In aqueous solutions the following reaction occurs;

B(C_6_H_5_)_4_^−^ + K^+^ ⇀ KB(C_6_H_5_)_4_↓ (s) (1)

The solubility of K-TPB and Na-TPB in water is 1.8 × 10^−4^ g/L and 470 g/L, respectively [36]. A 3 M stock solution of KCl was prepared by dissolving 11.175 g KCl in 50 mL ultrapure water and serially diluted to obtain a range of concentrations, quantified by AAS.

### 2.2. Sample Preparation for Generating a Calibration Curve

**Figure 1:**
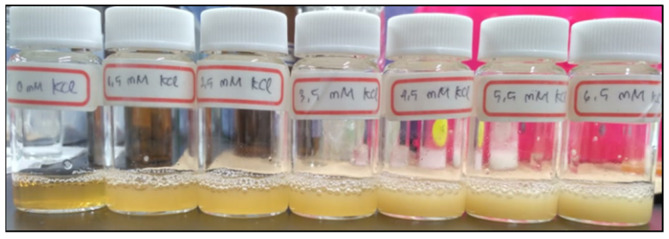
Turbidity of the treated blood plasma samples at different KCl concentration, in the presence of Na-TPB.

Each analytical method requires a reliable standard calibration curve. In ISE and AAS method, a calibration curve is obtained using aqueous solutions containing KCl standards. This way of calibration however does not include matrix effect that is important in real analytical application. Here, a calibration curve was generated on a treated blood plasma. The treated blood plasma was obtained in the following way. 12 mL of a fresh whole blood was centrifuged at 4 °C for 15 min at 2500 rpm, yielding 8 mL of blood plasma to which 4 mL of Na-TPB was added. The mixture was turbid, with a precipitate of K-TPB which was removed by centrifugation at 2500 rpm. Three cycles of precipitation were performed to remove all K^+^ from the blood plasma in supernatant. The obtained supernatant was regarded as K^+^-free blood plasma solution. A batch of samples for generating calibration curve were obtained by adding 100 μL of Na-TPB (146 mM) to 1.5 mL of this K^+^-free blood plasma. Subsequently, KCl was added to obtain samples with predetermined K^+^ concentrations (1.5–7.5 mM). The obtained samples could be characterized by different turbidity levels as shown in Figure 1.

To confirm the trend of turbidity changes, UV-VIS spectrophotometer was used to measure the transmittance value of each sample. The transmittance values were obtained under the following conditions: *λ_ex_* 260–800 nm; entrance slit width 5 µm; exit slit width 5 µm; scan speed 2 nm/s. A rectangular quartz cell (10 mm path, T-1-UV-10, JASCO, Tokyo, Japan) was used as the sample container. Each sample was diluted 60 times to adjust the transmittance value to a measurable level. 

### 2.3. Smartphone-Based Platform for Turbidity Measurements

**Figure 2:**
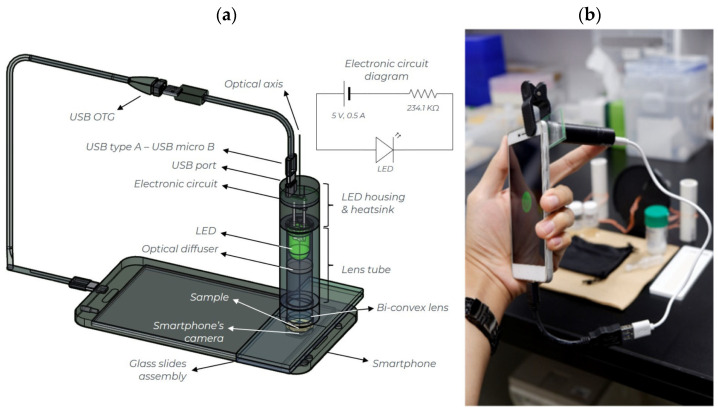
(**a**) Details of the optical attachment; (**b**) the attachment with the smartphone.

The smartphone-based platform consists of an optical attachment and a sample compartment as shown in Figure 2a. A Xiaomi Redmi 3 Pro smartphone was used in all experiments. It has a 13-megapixel camera with a sensor size of 4160 × 3120 pixels and a focal length of 4.22 mm. The images were recorded with the f/2.0 phase-detection auto-focus function. The optical attachment consists of a lens tube (length 40.9 mm, *Ø* = 12.7 mm; SM05L15, Thorlabs, New Jersey, USA) containing an optical diffuser (ground glass, N-BK7, *Ø* = 12.7 mm, 1500 grit, DG05-1500-MD, Thorlabs, New Jersey, USA) and a fixed bi-convex lens (N-BK7, *Ø* = 12.7 mm, f = 30.0 mm, LB1258-ML, Thorlabs, New Jersey, USA). The optical diffuser was used to spatially homogenize the LED intensity. The fixed bi-convex lens was used to shorten the focal distance of the camera, allowing it to focus on the diffuser over a short distance. The universal lens holder and moveable optical diffuser allow the optical attachment to be used with various smartphone cameras having different camera positions and focal distances. A commercially available green LED (*λ_max._* = 525 nm, 5 V, 300 mA, Akiba LED) was used as a light source. The LED intensity was adjusted to the level at which no saturation of the camera is observed. During experiments, the light intensity was around 0.29 μW and was controlled by a custom-built electronic circuit, shown in the inset of Figure 2a. The LED was mounted in an LED housing (LEDMT1F, Thorlabs, New Jersey, USA), which allows dissipation of the heat generated. The LED is powered by the smartphone via a USB on-the-go (USB-OTG) cable. The total cost of optical attachment excluding glass slide compartment is around $50.

The sample compartment is a single cavity glass slide (cavity 76 × 26 mm; Toshinriko Co., Ltd., Tokyo, Japan) and cover glass, as used for microscopic observation of biological samples. For each measurement, 15 µL blood plasma and 15 µL of Na-TPB solution was placed in the cavity. The cavity was closed with a cover glass (thickness 1.3 mm), sliding it across carefully to preclude air bubble formation. Between measurements the cavity was cleaned and a new cover glass slide was used. This glass slide sample compartment assembly was placed in the optical axis of the optical attachment as shown in Figure 2b.

### 2.4. Image Acquisition and Processing Algorithm

**Figure 3:**
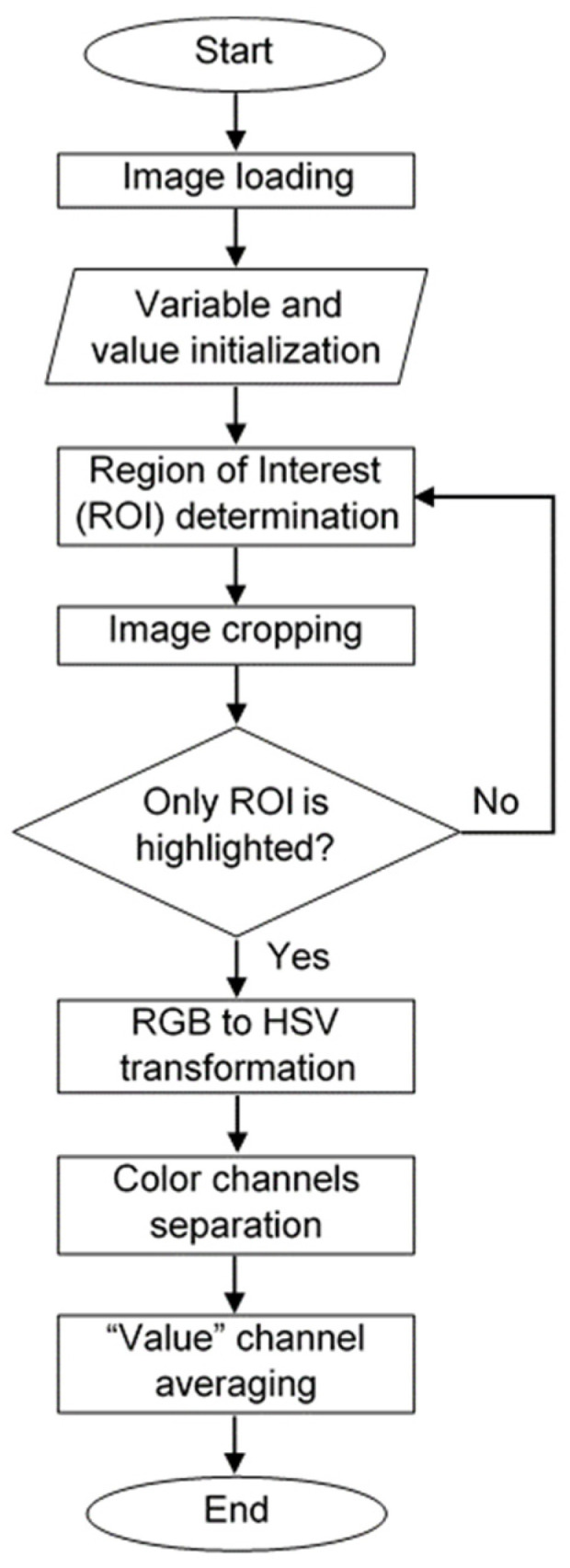
The image processing algorithm.

Images of samples containing different amount of K^+^ were taken with the following smartphone camera settings: manual, ISO 100, exposure time 1/12 s, aperture f/2.0. To quantify transmitted light intensity, images of the sample were analyzed using Matlab and a custom image-processing script written in C++ (Appendix A, Table A1). The image processing algorithm is shown in Figure 3. After loading the image into the Matlab application, various image properties, such as image dimension, color channels, and pixel data were assigned. Then, a region of interest (ROI) in the image was determined. The color channel of image in the ROI was then converted from the RGB (red, green, blue) to HSV (hue, saturation, value) color space. This conversion is useful to extract image intensity value. Readers interested in understanding color models in image processing are referred to review paper by Ibraheem et al. [37]. The mean ‘V’ pixel’s value of the image inside ROI was calculated and assigned as the transmitted light intensity. 

### 2.5. Details of ISE Device and AAS Instrument

A commercially available ISE device, LAQUA twin K-11 (Horiba, Japan), was used to evaluate the accuracy and precision of the smartphone-based method. The ISE device was calibrated prior to each measurement using standard aqueous solutions containing 150 ppm of K^+^ (Horiba, Japan).

A Thermo Scientific iCE 3500 AAS instrument was used that provides a wavelength range of 180–900 nm and can detect [K^+^] as low as 10 µg/L. In a typical measurement, a 20 mL of sample solution was prepared by diluting a 400 µL of blood sample in 19.6 mL ultrapure water. To generate a calibration curve, KCl concentrations of 0, 1, 2, 3, 4, 6, 7 mg/L were prepared by diluting the potassium standard solution TraceCERT with ultrapure water. Expected [K^+^] values in blood samples were derived from the obtained calibration curve.

## 3. Results and Discussions

### 3.1. Turbidity Measurement Using a UV-VIS Spectrophotometer

Initially, transmittance of the treated blood plasma solutions was measured using a conventional UV-VIS spectrophotometer (Supplementary material: Figures S1 and S2). No absorption peaks from blood components were found around the wavelength of the LED used in the smartphone optical attachment (525 nm). Thus, transmittance at 525 nm was used to quantify turbidity levels.

### 3.2. Turbidity Measurements Using the Smartphone-Based Platform

**Figure 4:**
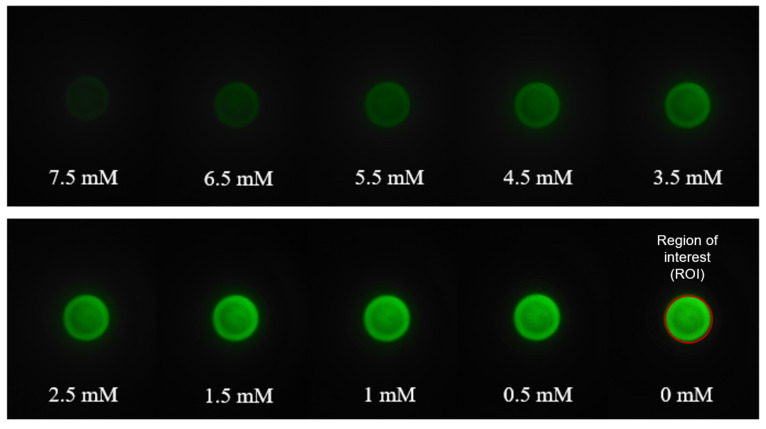
Images of blood plasma samples at different [K^+^], in the presence of Na-TPB.

Turbidity measurements were performed on blood plasma solutions containing the same amount of KCl as in the UV-VIS experiments. In each experiment 15 µL of the treated blood plasma and 15 µL of Na-TPB solution was loaded into the sample compartment. The sample compartment was then back-illuminated with 525 nm LED light. Since the LED light intensity is crucial for performing reproducible measurement, its stability was evaluated overtime as shown in Figure S3. The LED was allowed to be stable for two minutes’ prior each measurement. The light passing through the blood plasma solutions was captured by the smartphone camera. Images from blood plasma solutions containing different [K^+^] are shown in Figure 4. The intensity changes, captured in the range 0–7.5 mM K^+^ are clearly distinguishable.

### 3.3. Generating a Calibration Curve for the Smartphone-Based Method

**Figure 5:**
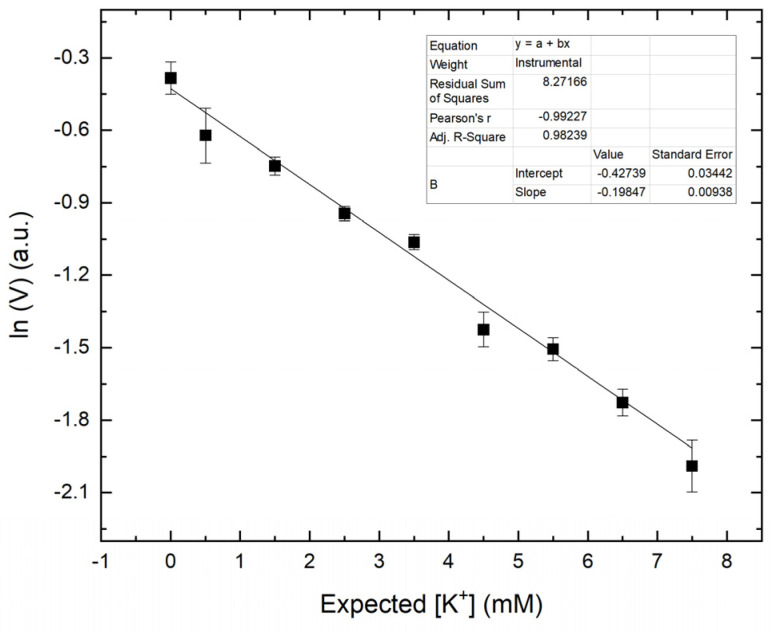
The ln of mean value component, V, for the treated blood plasma samples at given [K^+^].

A calibration curve was generated using the treated blood plasma solutions with predetermined [K^+^]. The samples were prepared according to the protocol described in Section 2.2. Images like the one shown in Figure 4 were analyzed using the Matlab script to obtain the mean value component (V) of the image inside ROI. In Figure 5, the ln of obtained mean V value for a given sample was plotted against the expected [K^+^].

Upon fitting the data, the following formula (2) was obtained and was applied to determine [K^+^] of test samples, of unknown [K^+^] in plasma.

(2) $$\left[K^{+}\right]=\frac{-\ln(V)-0.4274}{0.1985}$$

The results for a fresh bovine blood plasma test sample was 4.9 ± 0.11, consistent with the normal [K^+^] range in bovine blood which is 3.5–5.8 mM [38].

### 3.4. Accuracy, Precision, and Selectivity of the Smartphone-Based Method

Evaluation of accuracy and precision of this smartphone-based method was performed using controlled samples with known [K^+^]. A total of six samples at several concentrations within 0.5–7.5 mM were used. Each sample was measured six times and the mean [K^+^] was determined using the image-processing algorithm and used to generate a calibration curve (Figure 5). Above 1.5 mM, precision was within ± 0.27 mM and accuracy of reading was within ± 0.18 mM. The selectivity test of K^+^ over sodium cations (Na^+^) was conducted using sample of blood plasma containing 6.5 mM KCl and Na-TPB with additional presence of 125, 135, 145 and 155 mM NaCl. The results are shown in Figure S4. Among commonly coexisting ions in blood plasma, Na^+^ was chosen due to its highest concentration in blood plasma. The range of 125–155 mM NaCl was decided as it falls in the physiological concentration range. The results show that the presence of Na^+^ did not affect the K^+^ reading results. This is due to high selectivity of Na-TPB for K^+^ over Na^+^. Thus, the smartphone-based method is quite robust to determine potassium concentration with the presence of Na^+^ as an interfering ion in the blood plasma.

### 3.5. Comparison with ISE Device

**Figure 6:**
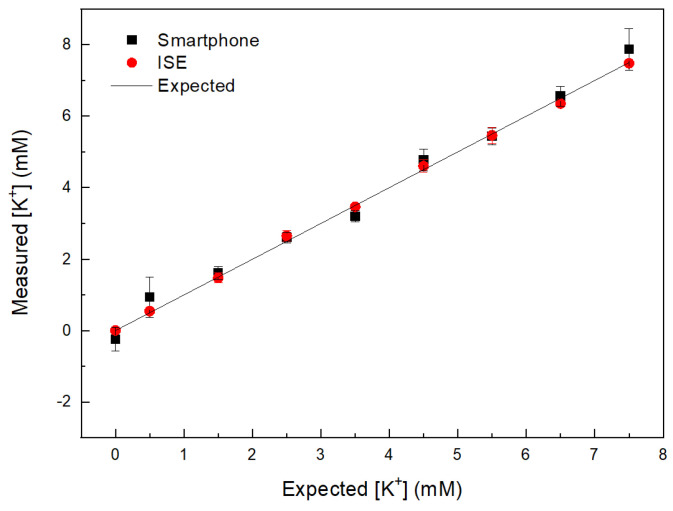
Plot of [K^+^] of the treated blood plasma samples obtained by the smartphone device and ISE device.

Figure 6 shows the measurement of [K^+^] using the smartphone device and ISE on blood plasma after adding known volume of KCl standard solution. The plot shows good correlation (R^2^ = 0.99) between data obtained using smartphone device and ISE device. Table 1 summarizes [K^+^] and errors obtained by each device.

A disadvantage of the ISE device is the frequent need for re-calibration due to accumulation of protein from blood plasma components on the surface of the electrode. When used for blood plasma samples, the electrode may quickly deteriorate and must be replaced frequently [39]. In contrast, in the smartphone device there is no direct contact between sample and sensor during measurements. In addition, ISE measurement requires ten times the sample volume used with the smartphone-based method which requires only 15 µL of blood plasma. 

### 3.6. Comparison with AAS

**Figure 7:**
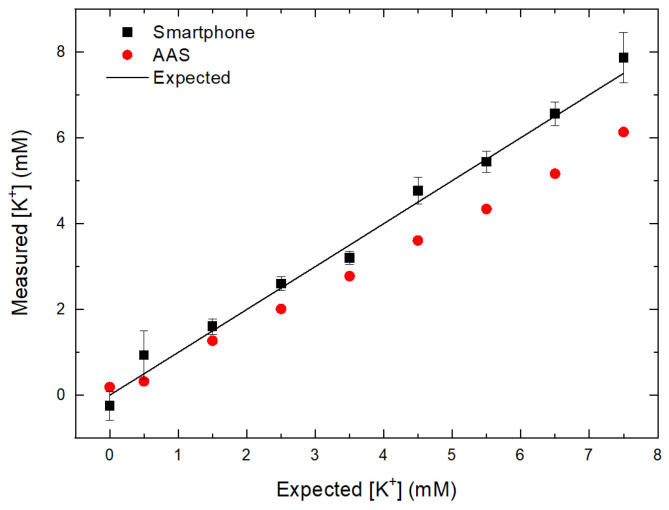
Plot of [K^+^] in blood samples obtained by smartphone device and AAS instrument.

Figure 7 shows the measurement of [K^+^] in blood plasma, after adding a known volume of KCl standard solution, using the smartphone device and AAS instrument. The measured [K^+^] corresponded fairly well with the expected [K^+^], but at higher concentrations, the measured values deviated from the expected values. This is most likely due to the inefficient process of atomization of solutions containing higher [K^+^] [40].

It is known that high salts concentration can lead to aggregation of protein in blood plasma by disruption of the hydration barriers between proteins [41]. The pH condition also affects aggregation behaviour [42]. Therefore, the flame condition of the AAS instrument should be adjusted for each sample separately to account for the increased mass. 

Another set of measurements (*n* = 25) was done on the lyophilized blood plasma with unknown [K^+^] and compared to results obtained for the same samples using AAS instrument. Lyophilized blood plasma was used due to logistic reasons and also to avoid uncontrollable K^+^ leakage from red and white cells to plasma during centrifuge process. A calibration curve for lyophilized blood plasma is shown in Figure S5. Table 2 summarizes the results.

[K^+^] obtained by smartphone device was close to the values obtained by AAS method. The lower [K^+^] in these samples is due to usage of lyophilized bovine plasma, in which loss of K^+^ might occur during sublimation and filtration processes [43]. This result shows that even with different source of bovine blood plasma, the method could give fairly accurate result for [K^+^] quantification.

## 4. Conclusions

The smartphone-based method developed in this study can be used to measure blood plasma [K^+^] with high accuracy in the range 1.5–7.5 mM. This method could be used in remote areas where access to a hospital is limited or by individuals who need to monitor daily potassium fluctuations. Blood separation may be achieved with a $2 hand-held centrifuge [44]. However, a calibration curve and its correlation function must be generated for each type of smartphone camera and plasma (fresh or lyophilized). After calibration, obtained correlation function could be stored in a dedicated Android application. With this application, the user could not only determine their blood plasma [K^+^], but also obtain associated online dietary and medical consultation, adding to the expansion of consumer products available from the IoT sector.

## Figures and Tables

**Table 1:** Comparison of [K^+^] in treated blood samples obtained with smartphone and ISE device.

[K^+^] (mM) Expected	[K^+^] (mM) Smartphone	[K^+^] (mM) ISE
0.5	0.93 ± 0.56	0.54 ± 0.05
1.5	1.60 ± 0.18	1.47 ± 0.14
2.5	2.59 ± 0.15	2.65 ± 0.15
3.5	3.19 ± 0.16	3.46 ± 0.03
4.5	4.70 ± 0.30	4.60 ± 0.17
5.5	5.44 ± 0.24	5.46 ± 0.22
6.5	6.56 ± 0.27	6.35 ± 0.09
7.5	7.86 ± 0.55	7.48 ± 0.08
Fresh [K^+^] plasma	4.90 ± 0.11	5.22 ± 0.09

**Table 2:** Comparison of [K^+^] of lyophilized blood plasma samples obtained by smartphone device and AAS.

Sample Number	[K^+^] (mM) Smartphone	[K^+^] (mM) AAS
1	2.21 ± 0.24	2.74 ± 0.001
2	2.69 ± 0.42	2.59 ± 0.007
3	2.76 ± 0.37	2.62 ± 0.005
4	2.94 ± 0.55	2.73 ± 0.009
5	2.42 ± 0.35	2.73 ± 0.008
6	2.52 ± 0.12	2.69 ± 0.007

## Data Availability

Not applicable.

## Supplementary Materials

The following are available online at https://www.mdpi.com/article/10.3390/s21144751/s1, Figure S1: Transmittance of blood plasma samples containing different KCl concentration, in the presence of Na-TPB, acquired with the commercial UV-VIS spectrophotometer, Figure S2: Transmittance value at 525 nm versus KCl concentration, Figure S3: LED light intensity plot overtime, Figure S4. Smartphone device reading results of blood plasma samples containing 6.5 KCl with additional presence of NaCl, Figure S5. The ln of the mean value component, V, for lyophilized blood plasma samples at given [K+].

## Appendix A

**Table A1:** Image-processing script.

Script	Comments
close all;	% closing all window
clear;	% clearing workspace
clc;	% clearing command window
I = imread (‘image’s file name’);	% loading image
imageSize = size (I);	% initializing image’s size
ci = [300,1534,2039];	% determining region of interest
[xx,yy] = ndgrid ((1:imageSize (1))-ci(1), (1:imageSize(2))-ci(2));	% generating image’s mask
mask = uint8((xx.^2 + yy.^2)<ci(3)^2);	
croppedImage = uint8(zeros(size(I)));	
croppedImage(:,:,1) = I(:,:,1).*mask;	% cropping Red-image with mask
croppedImage(:,:,2) = I(:,:,2).*mask;	% cropping Green-image with mask
croppedImage(:,:,3) = I(:,:,3).*mask;	% cropping Blue-image with mask
imshow(croppedImage);	% generating cropped image
hsvcroppedImage = rgb2hsv(croppedImage);	% transforming RGB to HSV image
h = hsvcroppedImage(:,:,1);	% Separating Hue-image
s = hsvcroppedImage(:,:,2);	% Separating Saturation-image
v = hsvcroppedImage(:,:,3);	% Separating Value-image
counted=sum(v(:)>0);	% Counting total spatial pixels
summedh_h=sum(h);	% Counting horizontal pixel value of Hue-image
summedh_s=sum(s);	% Counting horizontal pixel value of Saturation-image
summedh_v=sum(v);	% Counting horizontal pixel value of Value-image
summedv_h=sum(summedh_h,2);	% Counting vertical pixel value of Hue-image
summedv_s=sum(summedh_s,2);	% Counting vertical pixel value of Saturation-image
summedv_v=sum(summedh_v,2);	% Counting vertical pixel value of Value-image
avg_h=summedv_h/counted;	% Averaging “Hue” value across image
avg_s=summedv_s/counted;	% Averaging “Saturation” value across image
avg_v=summedv_v/counted;	% Averaging “Value” value across image
