# Supplementary material for: Smartphone-Enabled Quantification of Potassium in Blood Plasma
Source: Sensors (Basel). 2021 Jul 12;21(14):4751. doi: 10.3390/s21144751 (PMC8309773; doi:10.3390/s21144751)
Supplement: Supplementary file 1 [file sensors-21-04751-s001.zip › sensors-1261913-supplementary.pdf]

Supplementary Materials

Smartphone-enabled Quantification of Potassium in Blood Plasma

Achmad Syarif Hidayat [1], Hideyuki Horino [2], and Izabela I. Rzeznicka [1,*]

1 Graduate School of Engineering and Science, Shibaura Institute of Technology, 3-7-5 Koto-ku, Tokyo 135-8548, Japan; izabela@shibaura-it.ac.jp (I.R.); mg18501@shibaura-it.ac.jp (A.H.S.)

2 Tohoku University, 2-1-1 Katahira, Aoba-ku, Sendai 982-8577, Japan; hideyuki.horino.a6@tohoku.ac.jp

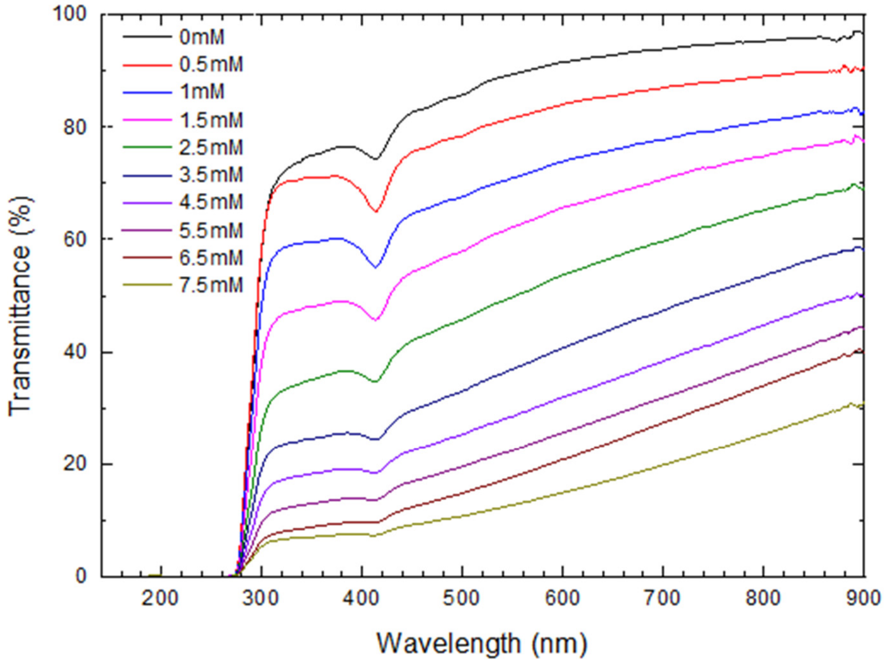

Figure S1. Transmittance of blood plasma samples containing different KCl concentration, in the presence of Na-TPB, acquired with the commercial UV-VIS spectrophotometer.

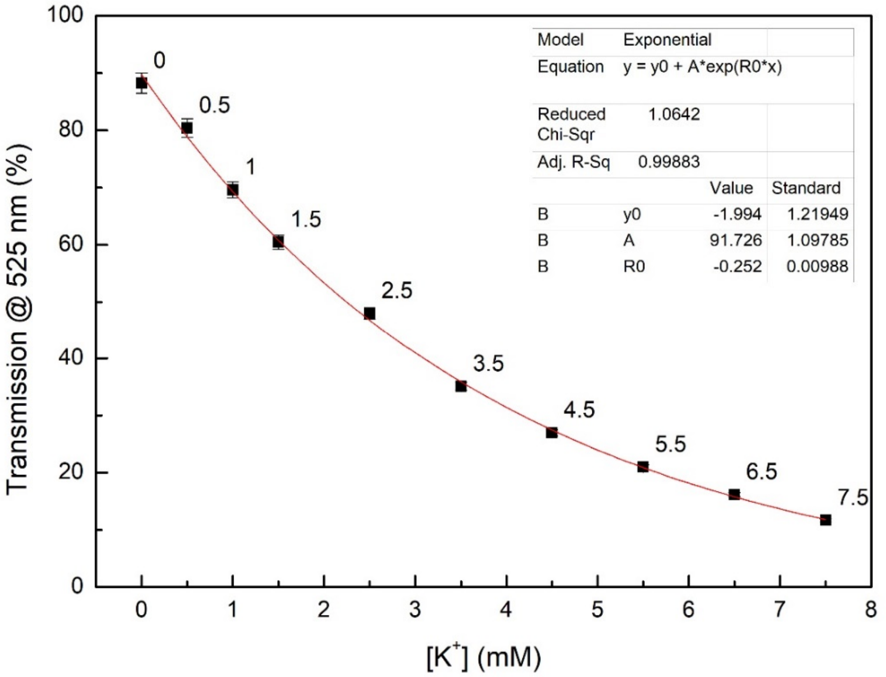

Figure S2. Transmittance value of the terated blood plasma at 525 nm versus KCl concentration.

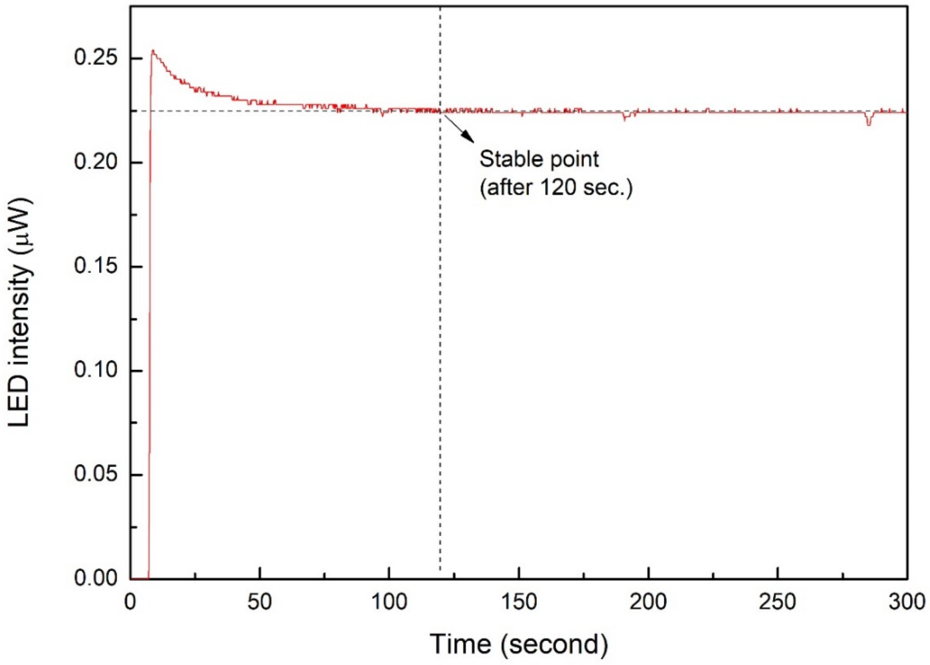

Figure S3. LED light intensity plot overtime.

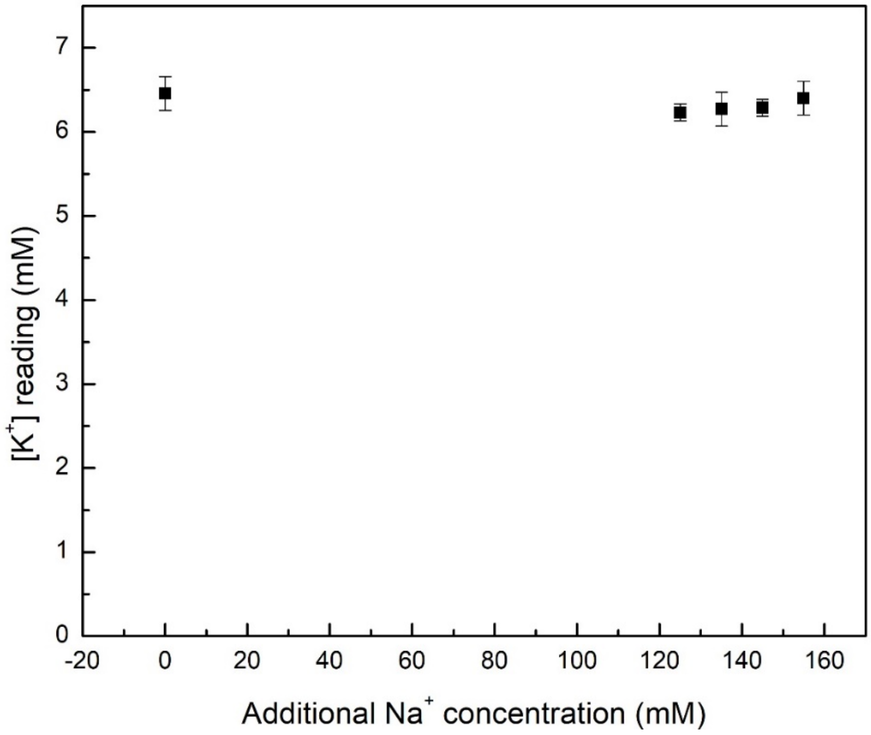

Figure S4. Smartphone device reading results of the treated blood plasma samples containing 6.5 KCl in the presence of NaCl.

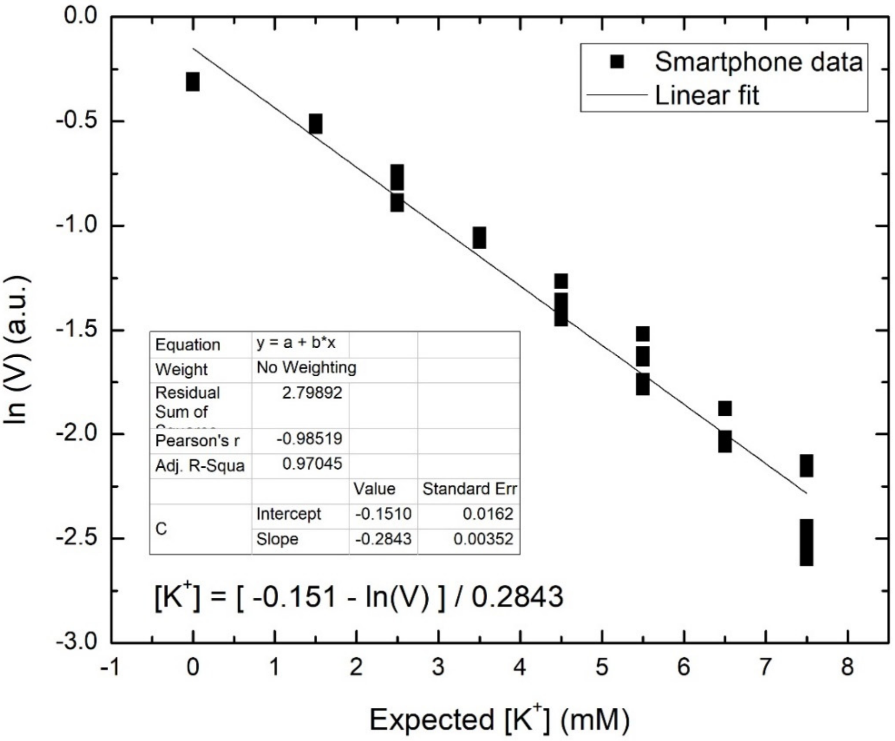

Figure S5. The ln of the mean value component, V, for lyophilized blood plasma samples at given $[K^+]$.
